# Trends in female breast cancer incidence, mortality, and survival in Austria, with focus on age, stage, and birth cohorts (1983–2017)

**DOI:** 10.1038/s41598-022-10560-x

**Published:** 2022-04-29

**Authors:** Lazo Ilic, Gerald Haidinger, Judit Simon, Monika Hackl, Eva Schernhammer, Kyriaki Papantoniou

**Affiliations:** 1 Department of Social and Preventive Medicine, Center for Public Health, Medical University of Vienna, Kinderspitalgasse 15, 1090 Vienna, Austria; 2 Department of Health Economics, Center for Public Health, Medical University of Vienna, Kinderspitalgasse 15, 1090 Vienna, Austria; 3 Austrian National Cancer Registry, Statistics Austria, Guglgasse 13, 1110 Vienna, Austria; 4 Department of Epidemiology, Center for Public Health, Medical University of Vienna, Kinderspitalgasse 15, 1090 Vienna, Austria

**Keywords:** Epidemiology, Breast cancer, Cancer epidemiology, Cancer prevention, Cancer screening

## Abstract

Breast cancer (BC) is the most commonly diagnosed malignant disease and the leading cause of cancer death in women in Austria. We investigated overall and subgroup-specific female breast cancer rates to provide a comprehensive analysis of trends over several decades. Incidence, mortality, and survival, as well as age-, stage-, and birth cohort-specific incidence were analysed using nationwide cancer registry data on 163,694 cases of female breast cancer in Austria (1983–2017). Annual percentage changes were estimated using joinpoint regression. BC incidence underwent linear increases until 1997 and reversed with statistically non-significant declines until 2017. After initial increases in BC-specific mortality, rates were stable from 1989 through 1995 and started declining thereafter, although statistically non-significantly after 2011. Overall BC-specific survivals, as well as survivals according to the calendar period of diagnosis, increased throughout the observation period. Incidence in younger women (aged 44 and lower) showed linear increases, whereas for women aged 45 and higher mostly stable or decreasing rates were observed. Localised BC incidence increased markedly and started declining only in 2012. Distant disease-BC incidence decreased through the whole observation period and incidence of regionalised BC started declining in 2000. Birth cohort-specific incidence peaked in women born between 1935 and 1949 (ages 45–74). In conclusion, the incidence of BC in younger women is increasing, while overall female BC incidence and mortality are stable with non-significant declines. Further, increases in the incidence of early-stage BC (localised) seem disproportionately high in comparison to more modest decreases in late-stage BC incidence (regionalised and distant disease).

## Introduction

Breast cancer accounts for a large part of the cancer burden in women worldwide and represented the most commonly diagnosed malignant disease and the leading cause of cancer-related death in 2020^1^. Correspondingly, breast cancer is leading in incidence and mortality among cancer sites in women in Austria^2^.

International breast cancer trends varied in the last decades as many high income-countries showed declines in female breast cancer mortality despite stable or increasing incidence rates, whereas increases in both mortality and incidence were observed in many lower- and middle-income countries^3^. In recent analyses of international breast cancer trends regarding menopausal status using data from 1998 through 2012, Austria stood out as an increase in premenopausal breast cancer incidence was accompanied by declines in postmenopausal incidence, a pattern only shared with the US and Israel^4^. Pre- and postmenopausal breast cancers differ regarding treatment options, risk factors, and survival rates as different molecular subtypes of female breast cancers show distinct age-specific incidence patterns^4,5^.

We conducted a comprehensive analysis of female breast cancer incidence, mortality, and breast cancer-specific survival in Austria between 1983 and 2017. Further, to discuss possible driving factors of breast cancer rates, we performed subgroup-specific analyses of incidence by age, stage of disease and birth cohort.

## Methods

### Data sources

Data on female breast cancer cases for the years 1983 to 2017 were obtained from the Austrian National Cancer Registry (ANCR), a nationwide cancer registry operated by Statistics Austria, based on Austria’s National Cancer Statistics Act of 1969. Austrian hospital departments report newly diagnosed cancer cases with a date of diagnosis, type and localisation of tumour or oncological disease, tumour stage at diagnosis, personal information of the patient, as well as details on hospital stay, diagnostic procedure and treatment. Further, the ANCR is linked to the Austrian Cause-of-Death Statistics, providing information extracted from death certificates. Compilation of data, data checks and quality assurance is performed by Statistics Austria according to requirements from the International Association of Cancer Registries^6^. Breast cancer diagnoses were coded using the International Classification of Diseases for Oncology Third Edition (ICD-O-3), with causes of death coded using the International Classification for Diseases 9^th^ and 10^th^ Revision (ICD-9 and ICD-10)^7–9^. Austrian population data were obtained from Statistics Austria, based on census data estimating the population on January 1st of each year. Mid-year population numbers were estimated and used as the denominator for the calculation of rates.

### Statistical analyses

All women diagnosed with breast cancer (coded using ICD-O-3) at an adult age as recorded in the ANCR between 1983 and 2017 were included in our analyses. Overall, age-adjusted mortality and incidence of breast cancer per 100,000 person-years were calculated by year of diagnosis. Overall breast cancer-specific mortality reflects all breast cancer cases regardless of subgroups (such as age, stage, and birth cohort) with a cause of death related to breast cancer. Age-adjusted incidence according to stage (per 100,000 person-years) was calculated using information on stage of disease at diagnosis (localised, regionalised, distant disease cancer, death certificate only-cases and unknown stages). Age groups were formed based on age at diagnosis (18–34 years, 35–44 years, 45–54 years, 55–74 years, and 75 + years). Birth cohorts were created by subtracting the age at diagnosis from the year of diagnosis providing up to 12 five-year birth cohorts per age group and a total of 22 five-year birth cohorts starting from 1885 through 1994 (1885–1889, 1890–1894, […], 1985–1989, 1990–1994). Calendar periods of diagnosis were created by grouping data on age at diagnosis in five-year groups starting with 1983–1987, 1988–1992, […] and finishing with 2013–2017.

Age-specific incidence was estimated by year of diagnosis and according to birth cohorts. Standardisation of rates was performed using the *distrate* command developed for Stata (Version IC 16.0), based on Tiwari et al.’s method with 95% confidence intervals estimated based on a gamma distribution^10,11^. Five-year and ten-year breast cancer-specific survival rates were extracted from lifetables calculated using Stata’s *ltable* command^12^. Individual-level survival in years was estimated by subtracting the year of diagnosis from the year of death and assuming an average survival of six months in cases of diagnosis and death reported in the same year. Time trend analysis was done using joinpoint models and the National Cancer Institute’s Joinpoint Trend Analysis Software (Desktop version 4.8.0.1.)^13^. In joinpoint models straight lines were fitted to the observed rates with joinpoints chosen and tested for significance using a Monte Carlo permutation method, providing information on trend changes (joinpoints) and annual percent change (APC), average annual percent change (AAPC) in rates between joinpoints^13^. The Joinpoint Regression Program provides users with confidence intervals relating to the nullhypothesis APC = 0 or AAPC = 0 as well as p-values indicating whether the null hypothesis of zero joinpoints (no trend changes) may be rejected. In certain cases, it is possible that the confidence intervals indicate significance whereas the p-values do not. In our study, we consider results significant if confidence intervals as well as p-values indicate significance, as seen in comparable studies^14^. The maximum number of joinpoints was set to four. The Bonferroni–Holm method was used to control for Type I errors.

### Sensitivity analysis

Preliminary analyses revealed increasing numbers of cases with missing information on stage of disease in more recent years. Full case- versus available case-comparisons showed a tendency of increasingly missing stage information in younger age groups (Appendix, Fig. A2). Given these findings and background information, such as the increased use of neo-adjuvant therapy leading to difficulties in accurate breast cancer stage definition, we concluded that data may not be missing at random or completely at random and refrained from confining to complete case-analysis and multiple imputation^15^.

### Ethics approval

Approval regarding ethical aspects of this study has been obtained by the Ethics Committee of the Medical University of Vienna (Study 1259/2019) in accordance with the Helsinki Declaration.

### Consent for publication

The data used for this study are part of the Austrian cancer registry and were compiled by Statistics Austria, Austria’s national statistics agency. The statutory collection of Austrian cancer registry data is performed without consent and is based on the 1969 Cancer Statistics Act and the 2019 Cancer Statistics Ordinance. The retrospective analysis and use of the fully anonymised data, as provided for this study, was approved by the Ethics Committee of the Medical University of Vienna.

## Results

A total of 163,694 breast cancer cases were recorded between 1983 and 2017 (Table 1). Breast cancer-specific deaths accounted for 32.5% of deaths in women with diagnoses of breast cancer. The median age at diagnosis was 64 years and the median age at death was 77 years (Table 1).

**Table 1:** Breast cancer incidence and deaths in women in Austria, Austrian Cancer Registry, 1983–2017.

Breast cancer incidence		Breast cancer deaths	
Total number of cases:	163,694*	Observed deaths among women with breast cancer (% of all cases):	88,096 (53.1)
		Breast cancer-specific deaths (% of all cases):	53,133 (32.5)

Information on diagnosis		Information on cause of death	
Coded using ICD-10:	100%*	Coded using ICD-10 (% of all deaths):	80,319 (91.2)
C500–C508:	12.7%	Coded using ICD-9 (% of all deaths):	7777 (8.9)
C509:	87.4%	Other cancer diagnoses (non C50*, % of all deaths):	7281 (8.3)
C809:	0.01%		

Age at diagnosis		Age at death (all deaths)	
Median (range):	64 (12–95 +)	Median (range):	77 (19–95 +)
Largest age group:	70–74	Largest age group:	70–74
Age < 50 (% of all cases):	33,133 (20.24)*		
Age ≥ 50 (% of all cases):	130,561 (79.76)		

*Cases in underaged (< 18 years of age at the time of diagnosis) were excluded (n = 12).

**Figure 1:**
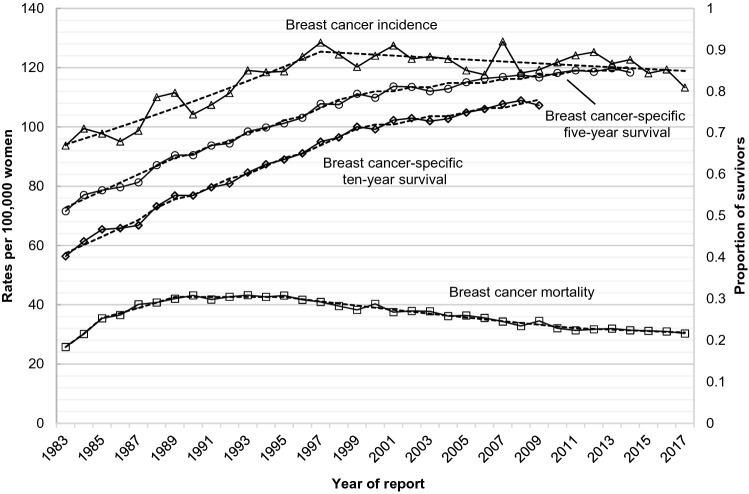
Overall incidence, breast cancer-specific mortality, and breast cancer-specific survival in women in Austria according to year of diagnosis, 1983–2017 (age-standardised, European Standard Population 2013). Dashed lines indicate joinpoint regression estimates.

**Figure 2:**
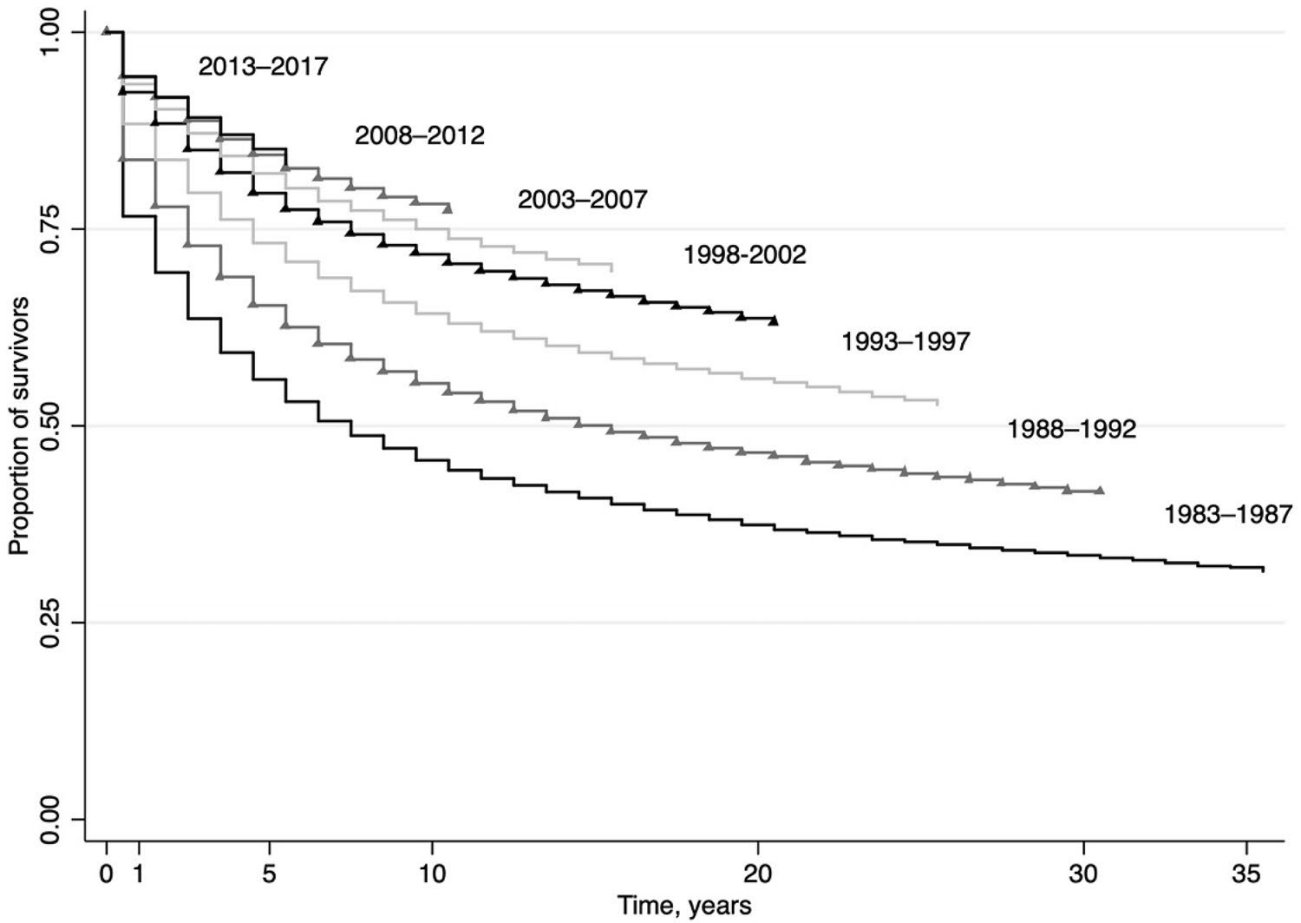
Kaplan–Meier estimates for breast-cancer specific survival in female patients in Austria according to period of diagnosis (five-year groups), 1983–2017.

Breast cancer incidence in Austria increased significantly from 1983 through 1997 (Table 2, Fig. 1). Incidence rates were stable and declining statistically non-significantly after 1997 through 2017. Mortality rates increased in two phases before 1989, were stable from 1989 to 1995 and decreased from 1995 on with statistically non-significant declines since 2011 as the most recent trend. Five-year and ten-year breast-cancer specific survival rates showed triphasic trends with considerable increases in survival from 1983 through 1989, followed by smaller increases in survival rates between 1989 and 1999 and further less pronounced improvements in survival after 1999 through 2014. Further, Kaplan–Meier estimates of breast cancer-specific survival according to calendar periods of diagnosis showed improving survival rates with each five-year group of observation (Fig. 2).

**Table 2:** Annual percent changes in overall breast cancer incidence, breast cancer-specific mortality, breast cancer-specific five-year and ten-year survival, and stage-specific and age-specific breast cancer incidence in women in Austria, 1983–2017.

	Years	APC %	(95% CI)
Overall incidence	1983–1997	**2.1**	**(1.6 to 2.5)**
	1997–2017	− 0.3	(− 0.5 to 0.0)
Overall breast cancer-specific mortality	1983–1985	**17.5**	**(8.8 to 26.9)**
	1985–1989	4.7	(1.4 to 8.1)
	1989–1995	0.0	(− 1.3 to 1.3)
	1995–2011	− **1.8**	**(− 2 to − 1.5)**
	2011–2017	− 0.8	(− 1.8 to 0.3)
Five-year breast cancer-specific survival	1983–1989	**3.5**	**(2.4 to 4.5)**
	1989–1999	**2.2**	**(1.8 to 2.6)**
	1999–2014	**0.5**	**(0.4 to 0.6)**
Ten-year breast cancer-specific survival	1983–1989	**4.5**	**(3.4 to 5.7)**
	1989–1999	**2.8**	**(2.4 to 3.2)**
	1999–2009	**0.9**	**(0.7 to 1.1)**
**Stage-specific incidence**			
Localised	1983–1997	**4.8**	**(4.3 to 5.3)**
	1997–2007	− 0.7	(− 1.4 to 0.1)
	2007–2012	3.4	(0.8 to 6.0)
	2012–2017	− 1.9	(− 3.6 to − 0.2)
Regionalised	1983–2000	1.1	(0.3 to 1.8)
	2000–2017	**− 2.4**	**(− 3 to − 1.7)**
Distant disease	1983–2017	**− 1.2**	**(− 1.6 to − 0.9)**
Unknown stage	1983–1997	**10.9**	**(8.0 to 13.8)**
	1997–2017	**2.4**	**(1.5 to 3.2)**
Death certificate only	1983–1996	**− 6.1**	**(− 7 to − 5.3)**
	1996–1999	23.0	(− 40.8 to 0.2)
	1999–2017	**− 1.8**	**(− 2.8 to − 0.9)**
**Age group (years)**			
18–34	1983–2017	**2.1**	**(1.6 to 2.5)**
35–44	1983–2017	**0.7**	**(0.5 to 0.9)**
45–54	1983–1993	**4.3**	**(3 to 5.6)**
	1993–2017	0.0	(− 0.2 to 0.3)
55–64	1983–2001	**2.6**	**(2.2 to 3.0)**
	2001–2017	**− 1.5**	**(− 2 to − 1.1)**
65–74	1983–2017	**0.8**	**(0.6 to 1.0)**
75 +	1983–1994	**2.1**	**(1.1 to 3.1)**
	1994–2017	**− 0.8**	**(− 1 to − 0.5)**

Bold indicates statistically significant results (*p* value < 0.05, corrected using the Bonferroni–Holm method).

*APC* annual percent change, *CI* confidence interval.

**Figure 3:**
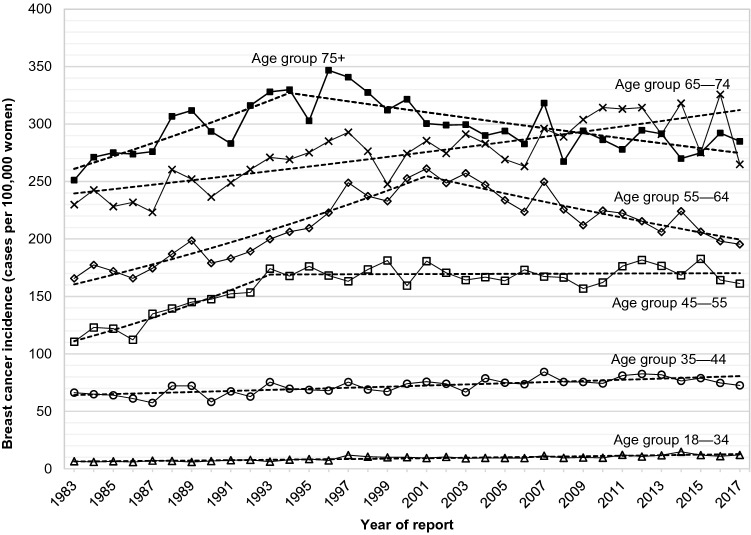
Age-specific incidence of breast cancer in women in Austria according to year of diagnosis, 1983–2017 (crude rates). Dashed lines indicate joinpoint regression estimates.

Breast cancer incidence trends among women in Austria varied markedly between age groups (Table 2, Fig. 3). Younger women (18–34 and 35–44 years) experienced increases in breast cancer incidence rates throughout the whole period of observation. Among women 45–54 years old, incidence rates increased from 1983 to 1993 and became stable after 1993 and through 2017. In the older age groups, trends were heterogenous. In 55–64 year old women and women older than 74 years, decreasing incidence rates were the most recent trend, whereas slowly increasing incidence rates were observed among 65–74 year old women over the whole observation period.

**Figure 4:**
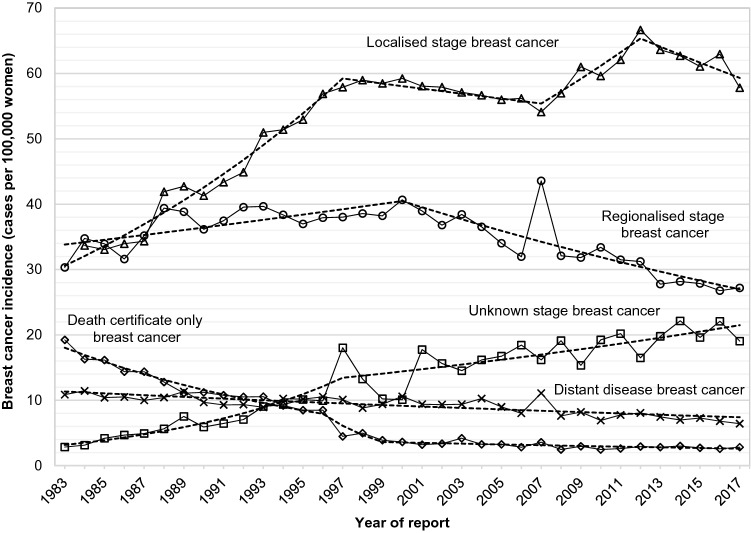
Stage-specific incidence of breast cancer in women in Austria according to year of diagnosis, 1983–2017 (age-standardised, European standard population 2013). Dashed lines indicate joinpoint regression estimates.

**Figure 5:**
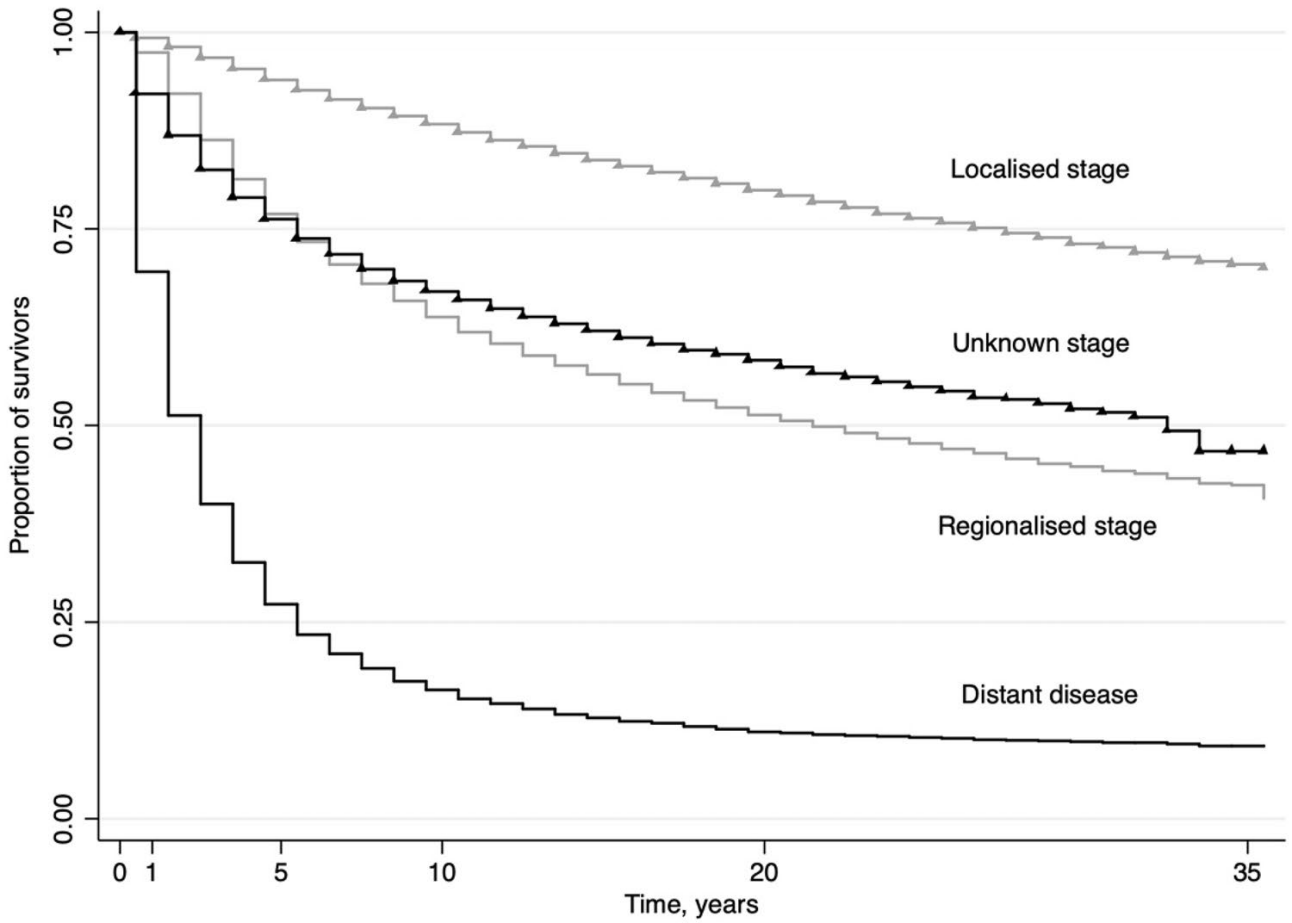
Kaplan–Meier estimates for breast-cancer specific survival in female patients in Austria according to stage at diagnosis 1983–2017.

Female breast cancer incidence trends in Austria varied markedly by stage (Table 2, Fig. 4). Incidence of localised breast cancer increased from 1983 until 1997 and was followed by more stable rates between 1997 and 2007, with a further increase in incidence from 2007 through 2012, followed by a trend reversion with a period of decrease until 2017 as the most recent trend (statistically non-significant). Regionalised breast cancer incidence increased from 1983 until 2000 (statistically non-significant) and was followed by a trend of stable decrease after 2000 and through 2017. Incidence of distant disease breast cancer in females in Austria showed a single trend of decreasing rates over the whole period of observation. Unknown stage female breast cancer incidence increased markedly until 1997, followed by a period of less pronounced increase until 2017. Kaplan–Meier estimates of breast cancer-specific survival according to stage at diagnosis showed decreasing survival rates from localised to regionalised and distant disease breast cancer, but also similar survival when comparing regionalised and unknown stage breasts cancer (Fig. 5).

**Figure 6:**
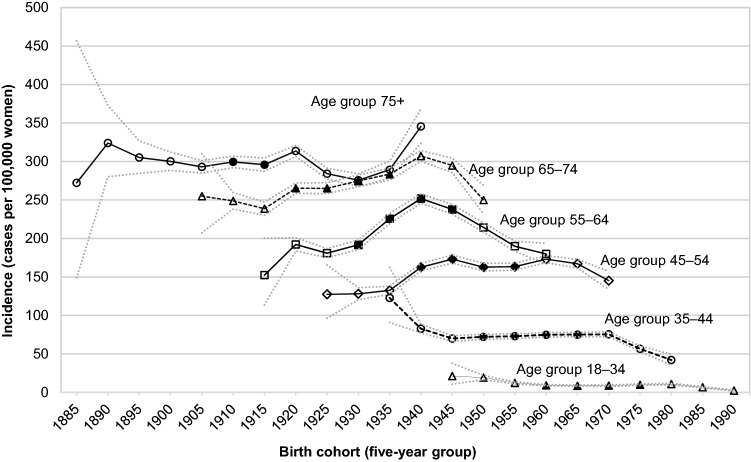
Age-specific incidence of breast cancer in Austrian females according to five-year birth cohorts, 1983–2017 (crude rates). Filled shapes indicate observations with fully captured age intervals without cut-offs due to exceeded observation limits (1983 and 2017). Dotted lines indicate 95% confidence intervals.

Figure 6 depicts age-specific incidence (five-year age groups) according to birth cohorts. Among women aged 45–54 years, 55–64 years and 65–74 years incidence peaked in the birth cohorts 1935–1940, 1940–1944, and 1945–1949. Younger women (18–34 years and 35–44 years) showed mostly stable incidence rates in the fully captured birth cohorts. Women in the oldest age group (75 years or older) showed high incidence rates throughout the whole observation.

## Discussion

Our study examined female breast cancer incidence, mortality, and survival changes over 34 years. Austrian women experienced a tendency of declines in overall incidence and mortality since 1997 and 1995 respectively as breast-cancer specific survival rates were improving. Subgroup-specific rates varied with increases in incidence observed in younger women (under 45 years of age) and stage-specific trends with disproportionately low reductions in more advanced stage cancer incidence as compared to the increases in early-stage breast cancer incidence between 1983 and 2017.

In previous analyses of data spanning from 1980 through 2002, Austria showed increases in breast cancer incidence between 1983 and 1996 and decreases in mortality between 1990 and 2002, changes similar to trends observed in Finland and Sweden during these periods^16^. In our data, the decreases in mortality in Austria started in 1995, whereas incidence shifted and started to decrease statistically borderline significantly from 1997 onwards. Further, our age-specific analyses are in accordance with and extend findings from an international comparison of breast cancer rates regarding menopausal status, where Austria stood out with increases in premenopausal breast cancer incidence (< 50 years of age) and declines in postmenopausal incidence between 1998 and 2012, a trend only shared with the US and Israel^4^. The declines in mortality starting after 1995 were preceded by breakthroughs in the treatment of breast cancer with the introduction of Tamoxifen in 1977 and Anthracyclines in the 1980s^17^. In parallel, substantial improvements in survivals were observed in the 1980s in Austria. Positive effects of secondary prevention through mammography screening may have played less of a role in the 1980s as uptake started to increase from 1995 on only^18^. Further, later improvements may be related to the centralisation of breast cancer management and the introduction of further treatment breakthroughs, such as taxanes (1994), trastuzumab (1998) and aromatase-inhibitors (2004)^17^.

Possible reasons for changing trends in breast cancer rates may come from changes related to individual-level risk factors, public health-related factors (such as screening), or improvements related to treatment and diagnosis of breast cancer. The simultaneous consideration of incidence, mortality, and survival or subgroup-specific trends may help hypothesise on the relative importance of suspected influencing factors and evaluate success of cancer control measures^19–21^. For instance, the positive effects of early detection through mammography screening may lead to increases in early-stage breast cancers and subsequent decreases in advanced-stage breast cancer and declines in mortality^20,21^. Decreases in mortality may also be a direct effect of improved treatment of breast cancer and may be reflected by increases in survival rates^22^.

In Austria, mandatory health care is provided to all citizens and the country’s health system is among the highest ranked in performance worldwide^23^. Thus, Austrian women likely were able to benefit from the major advancements in the treatment of breast cancer of the last decades. In terms of mammography screening, Austria went a different path as compared to countries with similar socioeconomic characteristics and health system performance, such as Sweden and Finland, where pilot projects of organised mammography screening date back several decades. In Austria, organised, invitational mammography screening was introduced only in 2014 and aimed at women aged 45 to 69^24^.

In our analyses, the increase in early-stage female breast cancer incidence in Austria (absolute change: + 29 cases per 100,000 women, 1983–2017) seems disproportionately high in comparison to the more modest decreases in late-stage breast cancer incidence (absolute changes: regional breast cancer − 4.8 cases per 100,000, distant disease breast cancer − 4.4 cases per 100,000).

These results resemble analyses of screening effects in the United States, suggesting overdiagnosis and limited success in reducing the incidence of advanced-stage breast cancers^20,21,25^. Some population-based studies failed to show effectual decreases in late-stage cases, suggesting limited effects of early detection, and raised concerns regarding overdiagnosis and questions about the extent of mortality reduction^20,21,25^. A further notable result from our analysis are decreases in localised stage breast cancer incidence between 2012 and 2017, despite the introduction of invitational screening in 2014. A previously active opportunistic, referral-based programme (established in 1972) showed increasing participation from the mid-1990s on with 81.9% in 2005^16,18^. Thus, the increases in localised breast cancer incidence before 2012, and decreases after 2012, may be a result of a “harvesting” effect from increasing mammography screening use from 1995 on.

In our age-specific analyses, younger women (18–34 and 35–44 years) experienced increasing breast cancer incidence over the whole period of observation. These findings are in accordance with recent research on global breast cancer rates regarding menopausal status with increasing incidence of premenopausal cancer (women younger than 50 years) in high-income countries^4^. Our results are also in line with studies reporting increases in breast cancer incidence particularly in young women (under 40 years of age) in Switzerland and the US^27,28^. A tendency of decreasing breast cancer-specific survival with younger age was attributed to increases in hormone receptor-positive high-grade cancer^26,27^. However, the possible reasons for the observed trends in younger age groups are manyfold, and may also reflect changes concerning modifiable risk factors^28^. Those may include factors associated with reproduction (e.g. higher age at first childbirth, decreasing per capita birth rate, reduced breastfeeding, hormonal contraception), occupation (e.g. shift work, work chemicals, long work hours, and deteriorating work-life balance), the environment (air pollution, environmental chemicals, etc.), diet, alcohol consumption, as well as smoking^28–31^. However, many of these risk factors are not exclusive to pre- or postmenopausal women and in the age groups above 45 years of age, we observed mostly stable or decreasing incidence rates, with the exception of 65–74-year-old women. The tendency of decreases in incidence rates in women > 45 years of age are a newly described trend shared with the United States (white population) and Israel (women > 50 years of age)^4^. Possible reasons for this are not apparent and might be a result of mammography screening and reduced hormone-replacement therapy (HRT) use since the early 2000s^4,32,33^.

Some cancer risk factors may not only be period-specific (such as the high prevalence of HRT-use peaking in the 1990s) but may also be birth cohort-specific such as smoking prevalence, fertility rates, body weight and reproductive factors in earlier birth cohorts. Our analyses of birth cohort-specific breast cancer rates in Austria showed lower incidence rates in earlier birth cohorts in the age groups 45–74. Peak-incidence rates for the 45–74 age groups were observed between 1935 and 1945. This may reflect changes in reproductive factors with decreased fertility in more recent cohorts, but also increased uptake of mammography or HRT-use in the late 1990s^18^.

In our study, we provide a comprehensive analysis of breast cancer rates over several decades using up-to-date data. However, a substantial increase of missing stage breast cancer cases in the analysed data (from 2.9 to 19 cases per 100,000, 1983–2017) introduces some uncertainty in the interpretation of the results of our analyses. In cancer registries with comparable reporting mechanisms to Austria, high numbers of missing stage information for breast cancer cases were reported^34,35^. Possible reasons are seen in the lack of information on the tumour stage at the time of diagnosis. Further, the increasing use of neoadjuvant therapy may introduce difficulties as the stage may differ before and after neoadjuvant therapy^34–36^.

Kaplan–Meier estimates of breast cancer-specific survival according to stage showed comparable survival patterns of regionalised and of unknown stage cancer (Fig. 5). Hypothetically, a part of the observed decreases in more advanced breast cancers may be resulting from increases in unknown stage breast cancer (Figs. 4 and 5).

A full case- versus available case-comparison based on age-groups analysis revealed larger numbers of cases with missing information on stage in younger age groups (Appendix, Fig. A2). These additional analyses leave the possibility of data missing not at random or completely at random unlikely, making the interpretation of stage-specific trends difficult^15^.

In conclusion, our comprehensive description of the Austrian breast cancer rates over several decades showed that although overall breast cancer incidence and mortality show tendencies of decline, new trends such as the increasing incidence of breast cancer in younger women are emerging. Stage-specific patterns hint at effects of advances in treatment with possible overdiagnosis by mammography screening. Further investigation of possible underlying factors for the observed increasing breast cancer incidence among younger women is necessary.

## Supplementary Information

Supplementary Figures.

## Data Availability

The data that support the findings of this study were provided by Statistics Austria. Restrictions apply to the availability of these data, which were used under license for the current study, and so are not publicly available. However, requests for the use of data may be made at Statistics Austria.

## Abbreviations

ANCR: Austrian National Cancer Registry (ANCR)
APC: Annual percent change (APC)
BC: Breast cancer (BC)
ICD-9: International Classification for Diseases 9th Revision
ICD-10: International Classification for Diseases 10th Revision
ICD-O-3: International Classification of Diseases for Oncology Third Edition
