# Author Correction: Trends in female breast cancer incidence, mortality, and survival in Austria, with focus on age, stage, and birth cohorts (1983–2017)

**DOI:** 10.1038/s41598-022-13018-2

**Published:** 2022-05-26

**Authors:** Lazo Ilic, Gerald Haidinger, Judit Simon, Monika Hackl, Eva Schernhammer, Kyriaki Papantoniou

**Affiliations:** 1 Department of Social and Preventive Medicine, Center for Public Health, Medical University of Vienna, Kinderspitalgasse 15, 1090 Vienna, Austria; 2 Department of Health Economics, Center for Public Health, Medical University of Vienna, Kinderspitalgasse 15, 1090 Vienna, Austria; 3 Austrian National Cancer Registry, Statistics Austria, Guglgasse 13, 1110 Vienna, Austria; 4 Department of Epidemiology, Center for Public Health, Medical University of Vienna, Kinderspitalgasse 15, 1090 Vienna, Austria

Correction to: *Scientific Reports* https://doi.org/10.1038/s41598-022-10560-x, published online 29 April 2022

The original version of this Article contained errors in the spelling of the authors Gerald Haidinger, Judit Simon, Monika Hackl, Eva Schernhammer & Kyriaki Papantoniou which were incorrectly given as G. Haidinger, J. Simon, M. Hackl, E. Schernhammer & K. Papantoniou respectively. The original Article has been corrected.

