# Supplementary material for: Trends in female breast cancer incidence, mortality, and survival in Austria, with focus on age, stage, and birth cohorts (1983–2017)
Source: Sci Rep. 2022 Apr 29;12:7048. doi: 10.1038/s41598-022-10560-x (PMC9054853; doi:10.1038/s41598-022-10560-x)
Supplement: Supplementary file 1 — Supplementary Figures. [file 41598_2022_10560_MOESM1_ESM.docx]

# Appendix

The following supplementary materials show the results of our sensitivity analyses yielding overall incidence rate ratios, overall breast cancer-specific mortality rate ratios, and age-specific incidence rate ratios based on full case- (exclude missing stage-cases) vs. available case-comparisons (include missing stage-cases), as depicted in Figure A1 and Figure A2. The analyses show increases in missing stage-cases when considering overall incidence (age-unspecific) and overall breast cancer-specific mortality (age-unspecific) (Figure A1). The results of the analysis on age-specific incidence rate ratios (Figure A2) suggest a tendency of larger increases in missing stage-cases in younger age groups.

Figure A1 Overall incidence and overall breast cancer-specific mortality rate ratios of breast cancer in women in Austria (1983–2017) in a full case- vs. available case-analysis


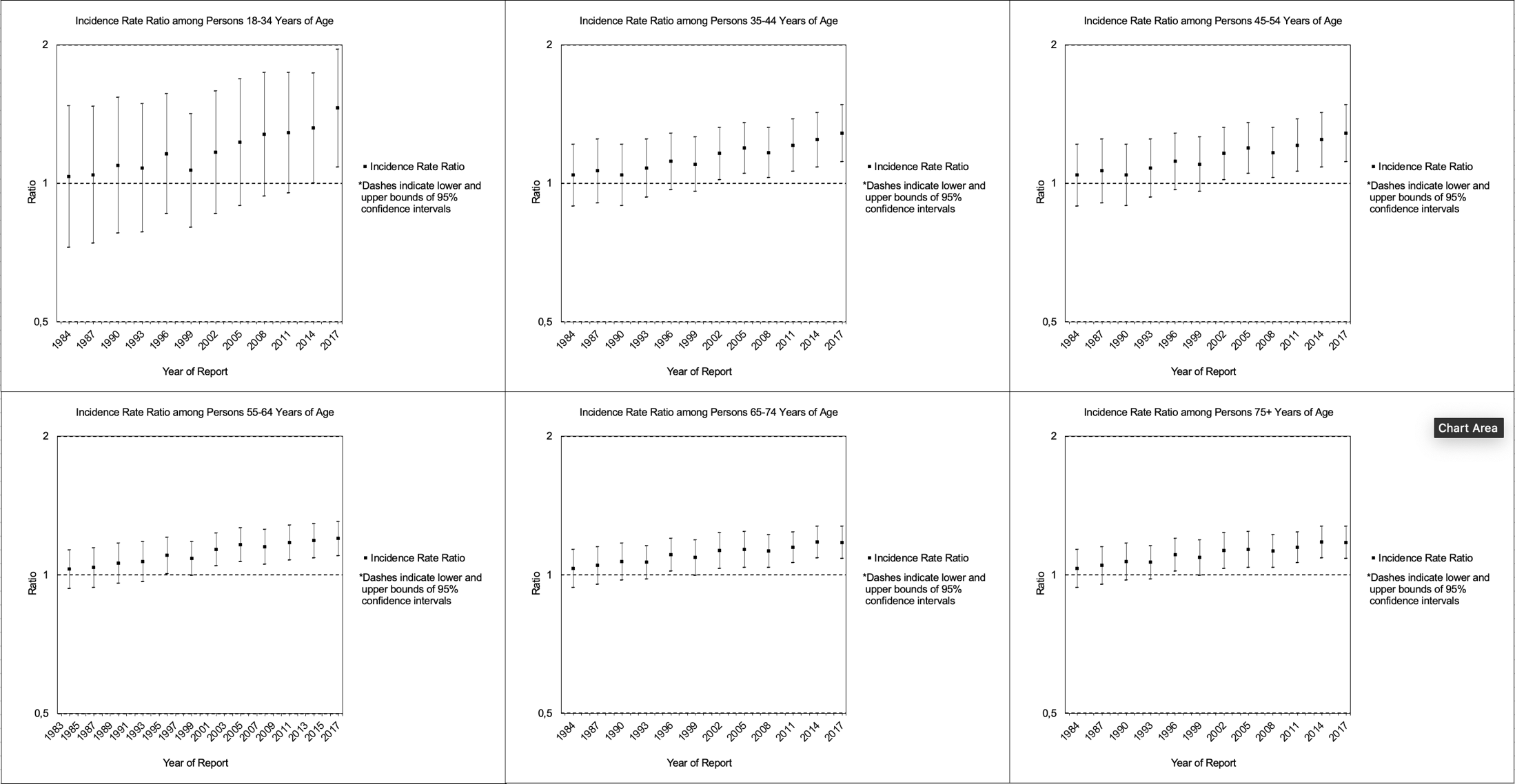


Figure A2 Age-specific incidence rate ratios of breast cancer in women in Austria (1983–2017) in a full case- vs. available case-analysis
